# Clinicopathologic features and surgical treatment prognosis of esophageal carcinosarcoma

**DOI:** 10.3389/fonc.2024.1387611

**Published:** 2024-08-21

**Authors:** Jiangfeng Shen, Kaijin Lu, Fuxing Liu, Xia Chen, Quan Chen, Bingbing Wu, Hailan Wang, Pengfei Ge, Guang Han, Fei Wang, Peng Zhang, Pei Yin, Weiguang Jia, Yiming Zheng, Pengcheng Wang, Fei Sun

**Affiliations:** ^1^ Department of Thoracic Surgery, The Affiliated Taizhou People’s Hospital of Nanjing Medical University, Taizhou, China; ^2^ Department of Pathology, The Affiliated Taizhou People’s Hospital of Nanjing Medical University, Taizhou, China

**Keywords:** esophageal cancer, carcinosarcoma, clinical pathology, surgical treatment, prognosis analysis

## Abstract

**Background:**

Carcinosarcoma is a rare esophageal tumor, accounting for approximately 0.27-2.8% of malignant esophageal tumors. This study aims to investigate the clinical pathological characteristics, surgical treatment outcomes, and analysis of prognostic factors in esophageal carcinosarcoma (ECS).

**Methods:**

Clinical data from sixteen patients diagnosed with esophageal sarcomatoid carcinoma who underwent surgical interventions were retrospectively analyzed. Clinical and pathological features, treatment modalities, and postoperative outcomes were systematically examined.

**Results:**

Out of the 1261 patients who underwent surgical treatment for esophageal cancer, 16 cases were pathologically confirmed as carcinosarcoma. Among them, two underwent neoadjuvant chemotherapy, six received postoperative chemotherapy. Carcinosarcomas predominantly occurred in the middle (43.75%) and lower (50%) segments of the esophagus. Among the 16 cases, 10 presented as polypoid, 4 as ulcerative, and 2 as medullary types. Microscopic examination revealed coexistence and transitional transitions between sarcomatous and carcinoma components. Pathological staging showed 5 cases in stage T1, 2 in stage T2, and 9 in stage T3, with lymph node metastasis observed in 8 cases (50%). TNM staging revealed 2 cases in stage I, 9 in stage II, and 5 in stage III. The overall 1, 3, and 5-year survival rates were 86.67%, 62.5%, and 57.14%, respectively. Univariate analysis indicated that pathological N staging influenced survival rates, while multivariate analysis demonstrated that pathological N staging was an independent prognostic factor.

**Conclusions:**

Carcinosarcoma is a rare esophageal tumor, accounting for approximately 0.27-2.8% of malignant esophageal tumors. Histologically, the biphasic pattern is a crucial diagnostic feature, although the carcinomatous component may not always be evident, especially in limited biopsies, leading to potential misclassification as pure sarcoma or squamous cell carcinoma. Despite its large volume and cellular atypia, carcinosarcoma carries a favorable prognosis. Complete surgical resection of the tumor and regional lymph node dissection is the preferred treatment approach for esophageal carcinosarcoma.

## 1 Introduction

Esophageal cancer ranks seventh in terms of incidence and sixth in mortality overall, which is responsible for one in every 18 cancer deaths in 2020. East Asia, especially China, has the highest incidence of esophageal cancer (1). The main risk factors contribute to the high incidence might include tobacco, alcohol, drinking hot beverages, low fruit and vegetable intake, consumption of unpiped water, and exposure to air pollution (2). Squamous cell carcinoma and adenocarcinoma account for more than 95% of all esophageal cancer cases worldwide. The remaining cases can be classified as rarely encountered histological subtypes such as small cell carcinoma, melanoma, choriocarcinoma, lymphoma, and sarcoma (3).

Carcinosarcoma, formerly known as spindle-cell, pseudosarcomatous, polypoid carcinoma, or sarcomatoid carcinoma, is a rare biphasic tumor distinguished by the concurrent presence of malignant epithelial and mesenchymal cell proliferations (4, 5). Due to its sarcomatoid component, esophageal carcinosarcoma (ECS) differs from ulcerative growth pattern in the other esophageal malignancies and are usually polypoid. Thus, the carcinomatous components of sarcomatoid carcinoma should be carefully identified to distinguish it from mesenchymal tumors (6). This study will describe specific clinicopathological and survival data for this group of patients.

## 2 Methods

### 2.1 Data source, patient selection, and variables studied

Retrospective case analysis was used in this study. A total of 1261 patients were diagnosed to have esophageal carcinoma based on histological evidence and underwent radical surgery at our institution from August 2015 to July 2023. After experienced pathologists reexamined tumor morphology and immunohistochemical results, 16 patients (1.27%) had a diagnosis of ECS and they were selected for inclusion in this study. The clinicopathological features, including location and primary tumor size, depth of invasion, disease status at diagnosis, and survival, were analyzed. Other variables analyzed included age, gender, smoking, alcohol consumption, and previous or concurrent history of cancer. The procedures in this study have been approved by the Ethics Committee of the Affiliated Taizhou People’s Hospital of Nanjing Medical University (No. KY-2023-188-01). All methods employed in this research adhere to the ethical principles derived from the Helsinki Declaration and its subsequent amendments, ensuring compliance with the standards set by the International Committee of Medical Journal Editors. Patients or their family members sign informed consent forms.

### 2.2 Inclusion criteria and exclusion criteria

Inclusion criteria: (1) age ≥18 years. (2) Histopathological examination confirmed esophageal cancer. (3) Preoperative evaluation of the tumor was resectable. (4) Completion of surgical treatment. (5) Clinicopathological data were complete.

Exclusion criteria: (1) Combined with other malignant tumors. (2) There is distant metastasis. (3) Lack of clinicopathological data.

### 2.3 Treatment methods

Neoadjuvant therapy combined with surgical treatment or direct surgical treatment was performed after discussion by the multidisciplinary diagnosis and treatment team according to the pathological type, stage and location of the patient’s tumor. According to the patient’s age, gender, tumor condition and preoperative examination results, the best surgical approach was selected according to the doctor’s experience: the surgical approach was divided into Mckeown esophagectomy, Ivor Lewis esophagectomy, and Transhiatal esophagectomy. McKeown Esophagectomy approach involves a three-incision esophagectomy: a laparotomy, a right thoracotomy, and a cervical incision. It allows for thorough lymph node dissection and is particularly useful for tumors located in the upper or middle esophagus. Ivor Lewis Esophagectomy procedure includes a laparotomy followed by a right thoracotomy. It is typically indicated for tumors located in the middle to lower esophagus. This approach facilitates a more extensive mediastinal lymph node dissection. Transhiatal Esophagectomy involves an abdominal incision and a cervical incision without thoracotomy. It is often chosen for tumors in the lower esophagus or gastroesophageal junction. This method reduces pulmonary complications associated with thoracotomy. We have utilized laparoscopic approaches to enhance precision and reduce recovery time. The choice of approach depends on the tumor’s location, stage, and the patient’s overall condition.

### 2.4 Immunohistochemistry

For Immunohistochemistry (IHC) analysis, formalin-fixed paraffin-embedded tissue specimens from each patient were sectioned into 4-μm slices. The mesenchymal component’s cellular differentiation in each ECS was characterized immunohistochemically using specific antibodies. Vimentin marked mesenchymal differentiation, smooth muscle actin (α-SMA) and desmin served as markers for muscle differentiation, while S100 protein indicated neural or chondroid differentiation. Immunoreactivity for each antibody was quantified by scoring the staining intensity (0, negative; 1+, weak; 2+, moderate; 3+, strong). The percentage of positive cells was calculated for each section without reference to clinical information. IHC positivity was defined when ≥5% of tumor cells showed moderate (2+) to strong (3+) staining. Additionally, Ki-67 expression was assessed to determine the proportion of proliferating cells. The Ki-67 labeling index (LI) was calculated as the percentage of Ki-67-positive nuclei among 1,000 tumor cells in each epithelial and mesenchymal component.

### 2.5 Observation indicators and evaluation criteria

Observation Indicators: (i) Clinical Pathological Characteristics: including patient gender, age of onset, pathological type, tumor location, pathological T (pT) stage, pathological N (pN) stage, TNM staging (International Union Against Cancer, UICC); (ii) Treatment Details: Neoadjuvant therapy, surgical approach, surgical margins, in-hospital mortality; (iii) Follow-up Information: Number of patients followed, follow-up duration, number of patients with follow-up >1 year, 1-year overall survival rate; (iv) Analysis of Prognostic Factors in Esophageal Cancer: Gender, age, tumor location, neoadjuvant therapy, surgical method, surgical margins, pT stage, pN stage, TNM stage.

Evaluation Criteria: Resection status categorized as R0 resection, R1 resection, R2 resection. Surgical death defined as death within 1 month postoperatively, and in-hospital death defined as death during hospitalization. Analysis limited to data from patients with follow-up >1 year. Tumor staging, lymph node staging, and prognostic staging based on the 9th edition of UICC TNM staging.

### 2.6 Follow-up

Follow-up conducted via telephone or outpatient visits, covering recent examination results, diet, and overall health status. In case of recurrence or metastasis, details such as time of occurrence, symptoms, treatment, and outcomes are recorded. Follow-up data collected until December 2023. Overall survival time defined from the date of surgery to death or the last follow-up. Survival and prognostic analysis performed for patients with follow-up >1 year.

### 2.7 Statistical analysis

Statistical analysis carried out using SPSS 24.0 software. Normally distributed continuous data presented as x ± s, and skewed data presented as M (range). Count data presented as absolute numbers. Kaplan-Meier method used for survival curve plotting and rate calculation. Log-Rank test employed for survival analysis. Log-Rank test for univariate analysis and COX regression model for multivariate analysis. Statistical significance set at *P*<0.05.

## 3 Results

### 3.1 Patient characteristics

In this study, 1261 patients were pathologically diagnosed with malignant tumors of the esophagus postoperatively, with no evidence of distant metastasis detected in preoperative examinations. Patients’ characteristics are shown in **Table 1**. The primary pathological types included squamous cell carcinoma (95.79%), carcinosarcoma (1.27%), small cell carcinoma (1.11%), neuroendocrine carcinoma (0.79%), adenosquamous carcinoma (0.4%), mucoepidermoid carcinoma (0.32%), epidermoid-like carcinoma (0.16%), and basaloid squamous carcinoma (0.16%). Regarding lymph node metastasis, the rates were as follows: squamous cell carcinoma (37.7%), carcinosarcoma (37.5%), small cell carcinoma (57.14%), neuroendocrine carcinoma (70%). In the I/II stages terms of TNM staging, carcinosarcoma (75%), squamous cell carcinoma (65.15%), small cell carcinoma (64.29%), neuroendocrine carcinoma (40%) were predominant. Carcinosarcoma exhibited a higher proportion in the early stages (I/II) compared to squamous cell carcinoma, small cell carcinoma, and neuroendocrine carcinoma.

**Table 1:** Histopathological Characteristics of 1261 Patients with Esophageal Malignant Tumors Undergoing Surgical Treatment.

Pathological feature	ESC	ECS	ESCC	ENC	EASC	EMEC	EEMC	EBSC
Gender								
Male	929	13	10	9	4	4	0	1
Female	279	3	4	1	1	0	2	1
T status								
Tis	25	0	0	0	0	0	0	0
T1	249	5	6	3	1	0	0	1
T2	198	2	3	1	2	1	1	1
T3	731	9	5	6	2	3	1	0
T4	0	0	0	0	0	0	0	0
Lymphatic metastasis								
None	753	10	6	3	4	4	0	2
Yes	455	6	8	7	1	0	2	0
Distant metastasis								
None	1208	16	14	10	5	4	2	2
Yes	0	0	0	0	0	0	0	0
TNM								
I	243	3	3	2	1	0	0	1
II	544	9	6	2	3	4	0	1
III	386	4	3	6	1	0	2	0
IV	35	0	2	0	0	0	0	0

ESC, Esophageal squamous carcinoma; ECS, Esophageal carcinosarcoma; ESCC, Esophageal small cell carcinoma; ENC, Esophageal neuroendocrine carcinoma; EASC, Esophageal adenosquamous carcinoma; EMEC, Esophageal mucoepidermoid carcinoma; EEMC, Esophageal epitheliomatoid carcinoma; EBSC, Esophageal basaloid squamous carcinoma.

### 3.2 Clinical and pathological characteristics

**Figure 1:**
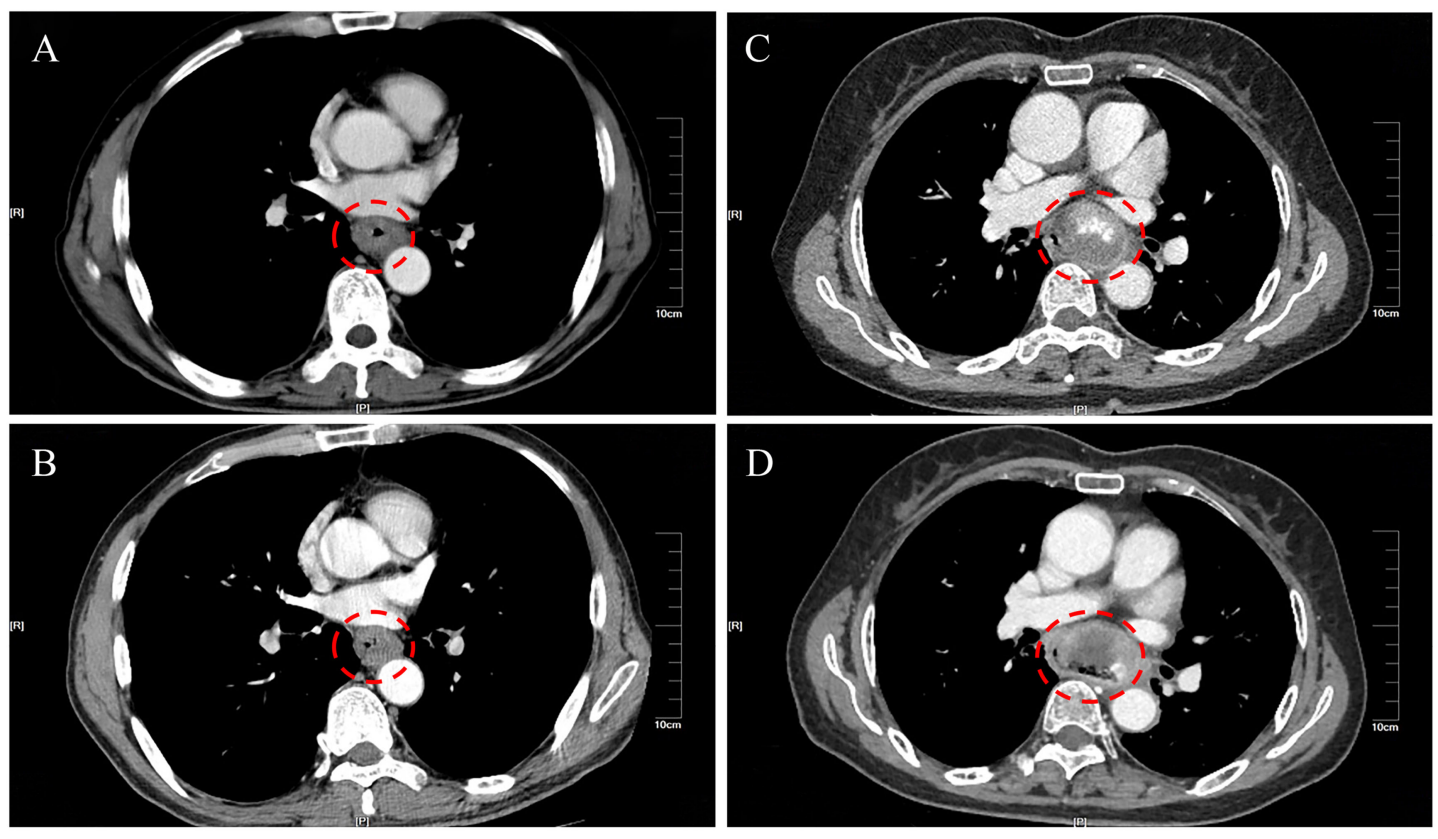
Comparison of thoracic enhanced CT scans of esophageal cancer tumors before and after neoadjuvant chemotherapy. **(A)** Case 3 before neoadjuvant treatment; **(B)** Case 3 after neoadjuvant treatment; **(C)** Case 10 before neoadjuvant treatment; **(D)** Case 10 after neoadjuvant treatment.

**Figure 2:**
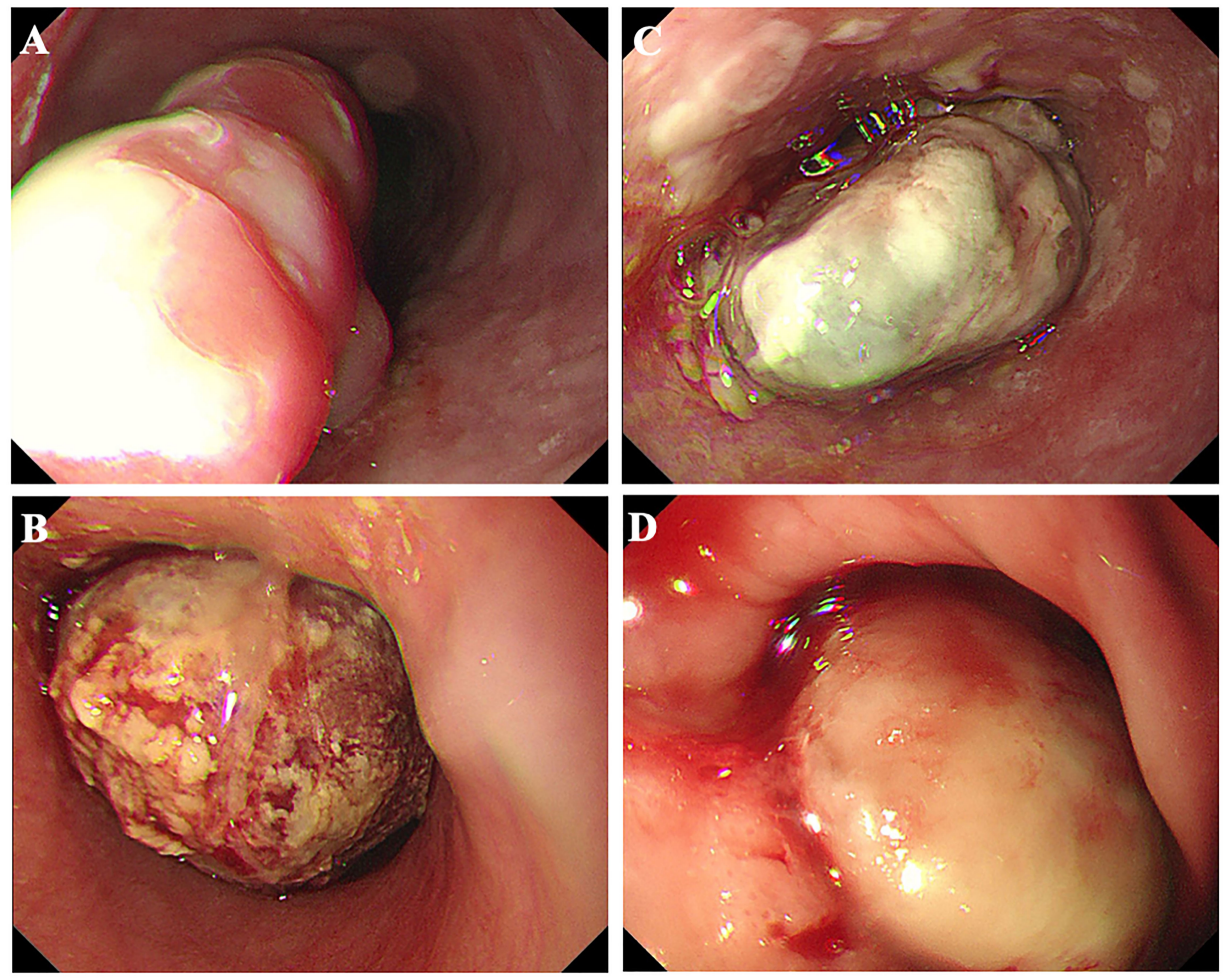
Esophageal carcinosarcoma patients commonly present with raised lesions during gastroscopy, characterized by a brittle texture and a propensity for bleeding. **(A)** Case 8; **(B)** Case 10; **(C)** Case 11; **(D)** Case 13.

All 16 patients in this study were pathologically confirmed as having ECS postoperatively. However, only 5 patients (31.25%) received a preoperative ECS diagnosis through biopsy pathology. Among the misdiagnosed cases through biopsy pathology, 62.5% were ultimately diagnosed as squamous cell carcinoma, while another case was diagnosed as a malignant tumor without a definitive pathological classification. The clinical and pathological characteristics of the 16 patients with ECS are summarized in **Table 2**. The majority were male (81.25%), with a median age of 68 years (range 54 to78 years). Tumor lengths were range 30 to 100 millimeter (mm), with half of the patients having lesions ≥50 mm and the other half <50 mm. Most tumors were located in the middle (43.75%) or lower thoracic (50%) of the esophagus, with fewer occurrences in the upper thoracic esophagus (6.25%). Two patients (case 3&10) underwent neoadjuvant chemotherapy with a combination regimen of docetaxel and cisplatin prior to surgery. However, after two cycles of treatment, there was insufficient evidence of tumor shrinkage. (**Figure 1**) Therefore, surgical intervention was pursued. Macroscopic evaluation revealed a polypoid morphology in 10 cases (62.5%), ulcerative morphology in 4 cases (25%), and medullary morphology in 2 cases (12.5%). (**Figure 2**) Microscopic examination identified concomitant squamous cell carcinoma and carcinosarcoma in 7 patients (43.75%), while 1 patient (6.25%) presented with a combination of squamous cell carcinoma, adenocarcinoma, and carcinosarcoma. Another patient (6.25%) presented with an esophageal tumor containing squamous cell carcinoma, signet-ring cell carcinoma, and sarcomatoid carcinoma components simultaneously. All patients exhibited spindle cell sarcoma components.

**Table 2:** Clinical finding and pathological features of the patients with ECS.

Demographics	n=	%
Sex		
Male	13	81.25
Female	3	18.75
Age, years		
≧ 65	12	75
≺ 65	4	25
Tumor size, mm		
≧ 50	7	43.75
≺ 50	9	56.25
Tumor location		
Upper esophagus	1	6.25
Middle esophagus	7	43.75
Lower esophagus	8	50
Neoadjuvant therapy		
None	14	87.5
Chemotherapy	2	12.5
Radiotherapy	0	0
Chemo/Radiotherapy	0	0
Treatment strategy		
Mckeown esophagectomy	4	25
Ivor Lewis esophagectomy	6	37.5
Transhiatal esophagectomy	6	37.5
Pathomorphology		
Polypoid	10	62.5
Ulcerative	4	25
Medullary	2	12.5
pTNM staging		
I/II	12	75
III/IV	4	25
Adjuvant therapy		
None	9	43.75
Chemotherapy	4	37.5
Radiotherapy	1	6.25
Chemo/Radiotherapy	2	12.5

### 3.3 Immunohistochemical expression

**Figure 3:**
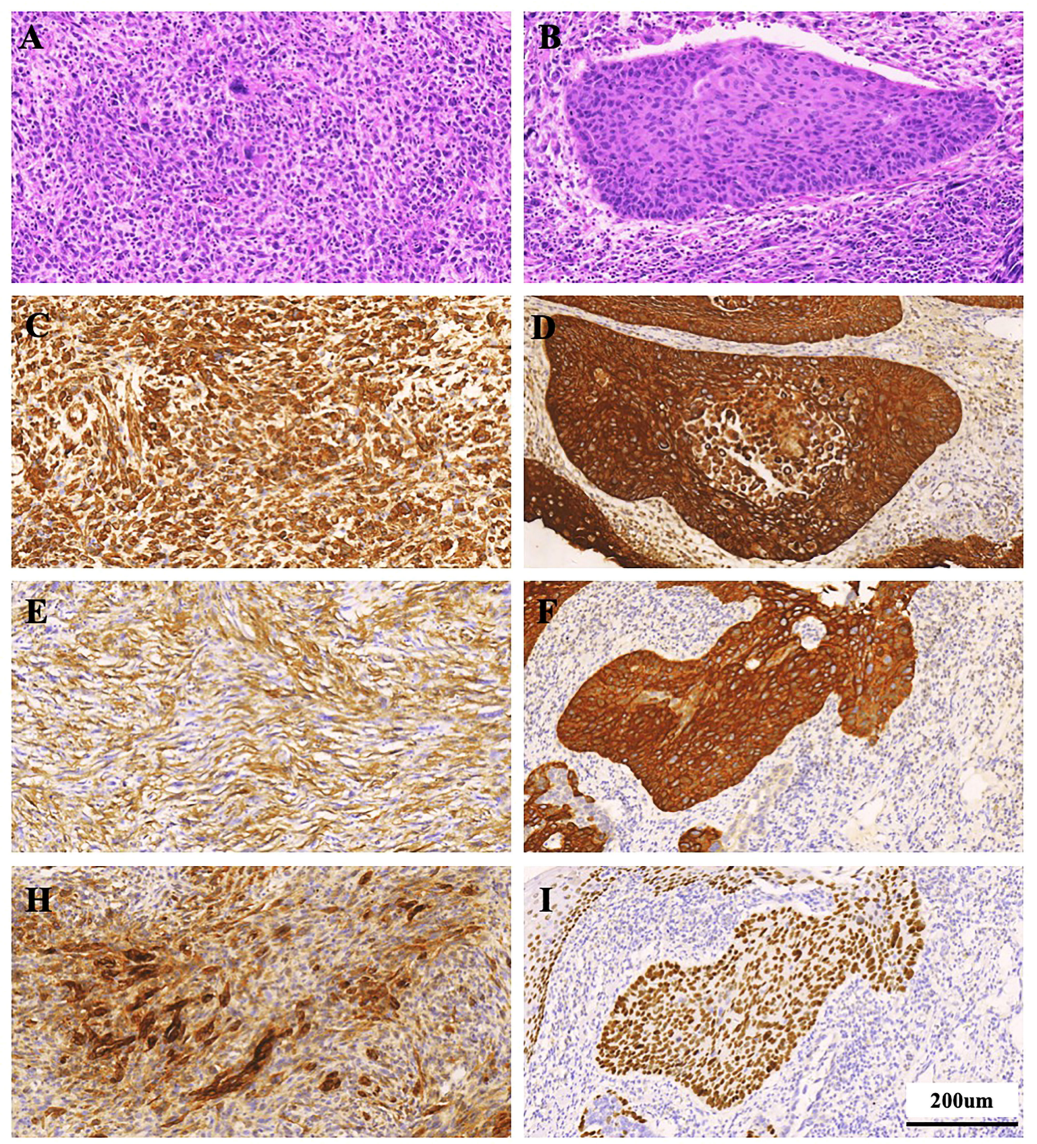
Histological staining observation of ECS patient (Case 11). **(A, B)** HE staining; **(C-I)** Immunohistochemical staining for Vimentin, CKpan, SMA, CK5/6, EMA, and P40. **(A, C, E, H)** Staining for sarcomatous component; **(B, D, F, I)** Staining for carcinoma component. Scale bars = 200 µm.

All histologically classified mesenchymal components showed immunohistochemical positivity for more than one mesenchymal marker. (**Figure 3**) Among the 16 cases of ECSs, vimentin, α-SMA, desmin, and S-100 were expressed in 15 (93.75%), 3 (18.75%), 0, and 0 cases of mesenchymal components, respectively. Epithelial markers CKpan, p40, p53, p63, CK5/6, and EMA exhibited positive expression in 11 (68.75%), 11 (68.75%), 13(81.25%), 12(75%), 13 (81.25%) and 4(25%) cases, respectively (**Table 3**).

**Table 3:** Immunohistochemical staining analysis.

Case	Vimentin	P40	P53	P63	CK5/6	Ckpan	Desmin	EMA	SMA	S-100	Ki-67
1	+		+++		+		–				70%
2	+++	++	+++		+	+++					90%
3	+	+++	+++	+++	+++	+++	–				70%
4	–	+++	+++	+++	+++			+++		–	50%
5	+++		++			+		–	+		60%
6	+	+	+	+	+	+		+	–	–	80%
7	++	+	+	+	+		–				35%
8	+++	+		+	+	+++	–				30%
9	+	+	+	+	+	+		+	++		70%
10	+++	–	+++	+++			–		+		70%
11	+++	+	–	+	+	+					40%
12	+	–	+++	+	+	+					80%
13	+	+	+++	+	+	+			–		60%
14	+	–	–	–	–	–		+		–	40%
15	+	+	++	+	+	+	–				65%
16	+	+	+++	+	+	+					70%

Immunoreactivity for each antibody was quantified by scoring the staining intensity (-, negative; +, weak; ++, moderate; 3+++, strong).

### 3.4 Treatment and complications

Sixteen patients underwent curative surgery, achieving complete tumor resection (R0). Among these patients, five (31.25%) unfortunately succumbed to various causes. One patient had a preoperative diagnosis of coronary artery disease. Unfortunately, this individual experienced sudden heart failure one month postoperatively, resulting in an unexpected demise. Consequently, this case was excluded from the final survival analysis. Another patient faced complications related to anastomotic fistula and passed away three months after surgery. The remaining three patients died due to tumor recurrence and metastasis within two years postoperatively, with the recurrence occurring between 10 and 23 months. On a more positive note, the other 11 patients (68.75%) are still alive (**Table 4**).

**Table 4:** Postoperative survival status of the patients with ECS.

Case	pTNM	Treatment^a^	Neoadju/adjuvant^b^	Status	Cause of death	Survival^c^
1	T3N0M0	Transhiatal	-/Chemo^d^	Alive	–	99
2	T3N1M0	Transhiatal	-/Chemo	Deceased	Recurrence	18
3	T3N2M0	Ivor Lewis	Chemo/Chemo + Radio^e^	Deceased	Recurrence	13
4	T1N0M0	Mckeown	-/-	Alive	–	74
5	T3N0M0	Transhiatal	-/-	Alive	–	73
6	T3N0M0	Transhiatal	-/-	Alive	–	73
7	T1N1M0	Mckeown	-/Radio	Deceased	Recurrence	10
8	T1N0M0	Ivor Lewis	-/-	Alive	–	38
9	T3N0M0	Transhiatal	-/-	Deceased	Anastomotic fistula	3
10	T3N0M0	Mckeown	Chemo/-	Deceased	Cardiac failure	1
11	T1N1M0	Ivor Lewis	-/-	Alive	–	31
12	T1N0M0	Ivor Lewis	-/-	Alive	–	21
13	T2N0M0	Ivor Lewis	-/Chemo + Radio	Alive	–	19
14	T2N0M0	Transhiatal	-/-	Alive	–	19
15	T3N1M0	Mckeown	-/Chemo	Alive	–	14
16	T3N1M0	Ivor Lewis	-/Chemo	Alive	–	13

^a^Treatment strategy; ^b^Neoadjuvant/adjuvant therapy; ^c^Survival of months; ^d^Chemotherapy; ^e^Radiotherapy.

Histopathological examination of all excised specimens revealed that 6 out of the 16 patients (37.5%) who underwent surgical treatment had lymph node metastasis. The most commonly affected nodes were those around the stomach (25%), followed by nodes adjacent to the esophagus (6.25%), cervical nodes (6.25%), and paratracheal nodes (6.25%). Lymph nodes around the trachea, inferior pulmonary veins, and pericardium were less frequently involved.

### 3.5 Follow-up information

Follow-up assessments were conducted regularly for all 16 patients postoperatively. As of the last follow-up, the median follow-up duration was 19 months, ranging from 1 to 99 months. Survival data for all 16 patients were obtained up to December 15, 2023. The survival rates for esophageal carcinosarcoma patients in this study were noteworthy. The 1-year, 3-year, and 5-year overall survival rates were 86.67%, 62.5%, and 57.1%, respectively. One patient exhibited remarkable survival, reaching 99 months post-curative esophagectomy without any signs of disease recurrence. These outcomes underscore the significance of careful and extended follow-up in monitoring the prognosis of patients with esophageal carcinosarcoma.

### 3.6 Analysis of prognostic factors in esophageal cancer

A single-factor survival analysis was conducted on 7 patients with a follow-up exceeding 5 years. The results revealed that the pathological N stage (χ² = 6.624, *P* = 0.01) significantly influences survival rates. However, factors such as gender, age (whether over 65 years), tumor length (whether over 50mm), tumor T stage, and pTNM stage showed no evident impact. Since patients undergoing neoadjuvant or postoperative chemoradiotherapy were generally in advanced stages, this factor was not included in the survival analysis. (**Table 5**) Multifactorial analysis similarly demonstrated that the pathological N stage (χ² = 5.844, *P* = 0.016) is an independent prognostic factor.

**Table 5:** Univariate survival analysis of esophageal carcinosarcoma with follow-up of 5 years.

Item	Cases	5-Year Survival Rate	χ² Value	*P* Value
Sex				
Male	7	57.14%		
Female	0			
Age, years			0.147	0.702
≧ 65	4	50%		
≺ 65	3	66.7%		
Tumor size, mm			0.24	0.624
≧ 50	2	50%		
≺ 50	5	60%		
T status			0.24	0.624
T1+T2	2	50%		
T3	5	60%		
N status			6.624	0.01
N0	4	100%		
N1	2	0		
N2	1	0		
pTNM staging			0.613	0.433
I/II	4	75%		
III/IV	3	33.3%		

Bolded indicate statistical significance.

## 4 Discussion

Esophageal cancer with pathological features divergent from squamous cell carcinoma or adenocarcinoma is an uncommon occurrence. The inclusion of esophagectomy in the treatment paradigm is warranted for the effective management of these rare types of malignant esophageal cancers (7). Carcinosarcoma is a rare malignancy of the esophagus, constituting approximately 0.27% to 2.8% of all malignant esophageal tumors (8–10). Among all surgically treated malignant esophageal tumors reported in our institution, the overall incidence of carcinosarcoma was 1.27% (16 cases out of 1261).

ECS encompasses both carcinomatous and sarcomatous components. Histologically, these two components are intermixed, with the sarcomatoid elements often prevailing. Additionally, there is a transition and migration between these two components (9). The pathogenesis of sarcomas has been elucidated through two primary explanations: (1) the theory of metaplasia and (2) the collision theory. The concept of metaplasia suggests that individual elements may originate from a common ancestral cell, giving rise to what is known as a sarcoma. On the other hand, the collision theory proposes that two separate stem cells may independently undergo malignant transformation, forming a genuine sarcoma (11). The treatment approach in this study was based on the fundamental principles derived from imaging, pathology, and the expertise of a multidisciplinary team (MDT), providing a more scientific and rational therapeutic strategy for this rare tumor type.

Okamoto and colleagues reported a rare case of esophageal carcinoma with sarcomatous and adenocarcinoma components (12). In our study, we identified a patient with an esophageal tumor containing squamous cell carcinoma, sarcomatoid carcinoma, and adenocarcinoma components simultaneously. The tumor measured 5.5×3×1.3 cm, staged as T1N0M0 (IB), and showed no recurrence or metastasis 21 months postoperatively. Another patient presented with an esophageal tumor containing squamous cell carcinoma, signet-ring cell carcinoma, and sarcomatoid carcinoma components simultaneously. The moderately differentiated squamous cell carcinoma exhibited a superficial elevated pattern, measuring approximately 3×3×0.5 cm. The sarcomatoid carcinoma region displayed a polypoid growth into the lumen, measuring 3×1×0.8 cm. The stage was T1N1M0 (IIB), and no recurrence or metastasis was observed 73 months postoperatively. To the best of our knowledge, this is the first report of the simultaneous occurrence of these three carcinoma components in a single patient.

ECS exhibits distinct biological characteristics compared to esophageal carcinoma: (1) it consistently displays a polypoid growth pattern; (2) metastatic lesions predominantly consist of pure sarcomatous components; (3) the prognosis is relatively favorable. The treatment of this condition does not differ from that of other malignant esophageal tumors. Early detection and diagnosis, followed by surgical resection, remain the primary strategies for promoting significant long-term survival in patients of this category (13). In our study, lymphadenectomy was tailored to the tumor’s location and stage: Two-field Lymphadenectomy typically performed for tumors located in the lower and middle esophagus, involving abdominal and mediastinal lymph nodes. Extended Two-field Lymphadenectomy includes additional lymph node dissection in the upper abdomen and lower mediastinum, often used for middle esophageal tumors. Three-field Lymphadenectomy was employed for upper and middle esophageal tumors, this comprehensive approach includes cervical, mediastinal, and abdominal lymph nodes. This method aims to improve survival rates by ensuring extensive lymph node clearance. Vagliasindi et al. (14) suggested that for surgical treatment of esophageal cancer with cervical and/or upper mediastinal metastatic lymph node involvement, three-field lymphadenectomy improves staging accuracy, enhances local disease control, and may improve patient survival, making it more suitable than two-field lymphadenectomy. Regarding lymph node metastasis, the rates were as follows: squamous cell carcinoma (37.7%), carcinosarcoma (50%), small cell carcinoma (57.14%), neuroendocrine carcinoma (70%). In the I/II stages terms of TNM staging, carcinosarcoma (68.75%), squamous cell carcinoma (65.15%), small cell carcinoma (64.29%), neuroendocrine carcinoma (40%) were predominant. Carcinosarcoma exhibited a higher proportion in the early stages (I/II) compared to squamous cell carcinoma, small cell carcinoma, and neuroendocrine carcinoma.

Immunohistochemical examination assumes a pivotal role in diagnosing ECS. The immunohistochemical characterization of the mesenchymal component in each ECS involved the use of specific antibodies: vimentin as an indicator of mesenchymal differentiation, smooth muscle actin (α-SMA) and desmin as markers of muscle differentiation, and S100 protein as a marker of neural or chondroid differentiation. The epithelial component typically exhibits positive expression of cytokeratin (CK), p40, p53, as well as p63, while epithelial membrane antigen (EMA) is also positively expressed. Furthermore, there are instances where epithelial tissue immunomarkers such as CK and EMA are also discernible in the sarcomatous component (15). It is noteworthy that, in some instances, the sarcomatous cells may exhibit cytokeratin staining, and the epithelial cells may stain positively for vimentin. Of significance, results of S-100 protein staining are negative, thereby distinguishing carcinosarcoma from stromal tumors (16). In our study, among the 16 cases of ECSs, vimentin, α-SMA, desmin, and S-100 were expressed in 15 (93.75%), 3 (18.75%), 0, and 0 cases of mesenchymal components, respectively. Epithelial markers CKpan, p40, p53, p63, CK5/6, and EMA exhibited positive expression in 11 (68.75%), 11 (68.75%), 13(81.25%), 12(75%), 13 (81.25%) and 4(25%) cases, respectively. The current findings underscore the utility of cytokeratin, predominantly expressed in epithelial tumor cells, and vimentin, consistently expressed in spindle tumor cells, as valuable biomarkers in the diagnosis of ECS (17).

The postoperative analysis of 16 patients in this study reveals the 1-year, 3-year and 5-year survival rates of 86.7%, 62.5% and 57.1%, respectively, better than the results reported by Kuo et al. in which the 1-year and 2-year survival rates of esophageal carcinosarcoma were 50 and 25%, respectively (8), and also better than that of reported by Wang et al. in which the overall 1-year, 3-year, and 5-year OS rates were 74, 57, and 48%, respectively (18). In comparison to the 5-year recurrence-free survival rate of patients with esophageal squamous cell carcinoma (ESCC) (less than 20%) (19), and that of patients with esophageal adenocarcinoma (EAC) (less than 20.1%) (20), the 5-year survival rate of patients with ECS was markedly elevated. Tumor recurrence manifested in 4 (25%) patients, predominantly within 2 years post-complete resection, with a median recurrence-free survival (RFS) of 26 months. Recurrences predominantly involved lymph node metastases and local relapses, which were managed with a combination of chemotherapy, radiotherapy, and in some cases, additional esophageal stent implantation. Despite these efforts, prognosis remained poor once recurrence was confirmed, highlighting the aggressive nature of esophageal sarcomatoid carcinoma. In a retrospective analysis conducted at a single institution, encompassing 28 patients who underwent esophagectomy for spindle cell carcinoma of the esophagus, Hashimoto et al. reported recurrence-free survival rates of 66.4% at 3 years and 61.6% at 5 years (21). The relatively favorable prognosis of ECS may be attributed to the polypoid nature of the lesion. The tumor typically exhibits an exophytic growth pattern towards the lumen, with infiltration depth often limited to the layers of the esophageal wall, including the muscle layer, and may not extensively involve the outermost serosal layer (10). Hence, individuals presenting with early-stage dysphagia symptoms tend to exhibit more favorable survival outcomes compared to patients with conventional esophageal cancer of equivalent size (18).

Certain research findings are inconsistent with the conclusion of ECS having a favorable prognosis, possibly due to a relatively higher proportion of lymph node metastasis in the study population (22). ECS represents a distinctive form of esophageal malignancy. Our investigation reveals a substantial elevation in the risk of lymph node metastasis following ECS invasion of the esophageal muscle layer. These results underscore the crucial significance of radical esophagectomy combined with lymph node dissection. Preoperative neoadjuvant chemotherapy is not effective for esophageal carcinosarcoma. In this study, two patients received two cycles of neoadjuvant chemotherapy with docetaxel combined with cisplatin. However, the esophageal tumors did not significantly shrink, and there was no apparent benefit in terms of prognosis (with survival periods not exceeding 2 years in both cases). Presently, surgery stands out as the foremost therapeutic approach and should be regarded as the primary intervention for ECS patients in a stage-appropriate manner (23).

The conclusions drawn in this study should be considered with certain limitations. Firstly, it is a retrospective analysis, and there is a possibility of incomplete or inconsistent data. Subsequent studies could benefit from prospective designs. Secondly, the sample size in this study is relatively small, and future research should consider expanding the sample size appropriately. Lastly, the follow-up period in this study is not extensive, and there is a need for a more prolonged follow-up duration in future investigations.

## 5 Conclusions

Carcinosarcoma is a rare esophageal tumor, accounting for approximately 0.27-2.8% of malignant esophageal tumors. Its origin and nature remain unclear, and clinically and radiologically, it shares similarities with other malignant esophageal tumors. Histologically, the biphasic pattern is a crucial diagnostic feature, although the carcinomatous component may not always be evident, especially in limited biopsies, leading to potential misclassification as pure sarcoma or squamous cell carcinoma. Despite its large volume and cellular atypia, carcinosarcoma carries a favorable prognosis. Complete surgical resection of the tumor and regional lymph node dissection is the preferred treatment approach for esophageal carcinosarcoma.

## Data Availability

The original contributions presented in the study are included in the article/supplementary material. Further inquiries can be directed to the corresponding author.
